# Posthuman interventions in submerged histories: reconstructing history through memory in Rivers Solomon’s *The Deep*

**DOI:** 10.3389/fsoc.2025.1612388

**Published:** 2025-08-25

**Authors:** Abhishika Dawn, G. Alan

**Affiliations:** Vellore Institute of Technology (VIT), Chennai, India

**Keywords:** posthuman, postmemory, counter-memory, catharsis, slave trade

## Abstract

A community’s collective memory is predominantly shaped by dominant power structures that generate and contain canonical narratives. Within the post-colonial context, this social memory remains in conflict with certain ancestral or tribal memories that witnessed the violent legacies of colonization. These memories, which are transmitted across generations—termed postmemory—aims to reclaim and expose the officially silenced histories through the production of counter-memory. Rivers Solomon’s *The Deep* (2019) explores the historical injustices inflicted upon the African community during the transatlantic slave trade, their impact on successive generations, and the production of counter-memories as a means of resistance and collective catharsis. Solomon crafts a narrative populated by posthuman characters, who in a life-death continuum, embody postmemories and transform their suffering into resistance through the articulation of counter-memories. Through the concept of postmemory, Solomon engages in the process of mourning, simultaneously incorporating counter-memory to call attention to what has been erased from the collective narratives. The paper also seeks to explore the creative reworkings of historical events through posthuman figurations that defy the normative power location of the subject position, highlighting how these concepts intersect to facilitate new avenues for understanding collective experiences of trauma and resilience in the face of systemic erasure.

## 1 Introduction

Official historical records are produced by dominant groups who create and maintain unity by imposing a singular interpretation of the past, effectively marginalizing alternative interpretations of the same historical experiences. Several historical interpretations, explanations, and descriptions have been biased as they align with the historian’s interests (McCullagh, 2000, p. 39). For example, the histories of displaced Muslims and Sikhs during the partition of India, the Vietnamese during the Vietnam War, and the African communities during the slave trade, among others, have long been silenced or erased from mainstream historical discourse. Consequently, these histories, which were omitted from official history, are kept alive through postmemories—the transmission of discourses and traumatic events to the next generation. This “received, transferred knowledge of events is being transmuted into history, or into myth” (Hoffman, 2004, p. xv) and tends to endure even after the participants are gone (Hirsch, 2008, p. 111). The postgeneration begins to assert their own victimhood alongside that of the parents (Hirsch, 2008, p. 112). In this context, the historical and emotional weight of the postmemories produces a counter-perspective that challenges and resists the prevailing dominant ideology. Thus, counter-memory has the potential to supply new perspectives about past events and revise existing histories for those who were denied adequate historical representation.

In the posthuman paradigm, memory is conceptualized as a communal and timeless construct rather than a personal one. It “works in terms of nomadic transpositions, that is to say, as creative and highly generative inter-connections which mix and match, mingle and multiply the possibilities of expansion and relations among different units or entities” (Braidotti, 2006). Posthumanism challenges the idea of memory being solely tied to the human mind or consciousness (Critical Posthumanism, 2024). In the context of coexisting with both humans and nonhumans, posthumanism questions anthropocentric biases by foregrounding the non-anthropocentric (Gündoğan İbrişim, 2024, p. 94). In pursuit of this stance on memory, the novella *The Deep* rethinks the African slave trade within a posthumanist context by centering on a posthuman subject—a nonhuman female called wajinru. This defamiliarizes us from the dominant vision of the self that one is accustomed to and evolves into a posthuman framework. Through the vulnerability of the enslaved humans, the novella maps itself into the existence and vulnerability of the nonhuman other as well. This presents a way of living, interacting, and remembering in an eco-ethical manner that prompts readers to reconsider the ways in which memory can be a shared experience that transcends species boundaries, acknowledging the multiplicity of otherness on the changing planet.

The novella *The Deep* narrates the lives of the African slave descendants and their forced migration during the transatlantic slave trade. A particularly harrowing aspect of this history is the practice of discarding sick or injured slaves (including pregnant slaves) as they were deemed unwanted cargo. The narrative centers on the descendants of these deceased mothers who are now consigned to the sea, becoming “aquatically mutated descendants of those unfortunate victims of human greed” (Solomon, 2019, p. 106), known as wajinru. Within this underwater society, the hybrid half-human and half-fish wajinru are divided into two categories: historian and subjects. Because of the intrinsic trauma in their history, only the historian is tasked to hold the rememberings of the wajinru community. The character Yetu is bestowed with the sacred role of a historian, who bears the weight of preserving the community’s collective memories, encompassing the harrowing experiences and trauma associated with their past. While the rest of the community forgets the painful memories of their past over time, Yetu’s role compels her to retain and recount every bit of it and annually facilitate the other wajinru to experience their histories. During the annual ceremony of Remembering, Yetu allows the other wajinru to temporarily experience their history long enough for them to regain their sense of belonging. Once they get their fill, sufficient to fuel them for 1 year, the history is returned to Yetu. This psychological toll of her role, along with the complexities of remembering a collective history fraught with suffering, disorients her. During one such annual ceremony, when the history is temporarily taken away from her to give to the other wajinru, she experiences a moment of lightness and liberation. In a desperate instinct for self-preservation, she decides to escape without retrieving the history. During this journey away, she rediscovers the human world that her community had long forgotten.

The novella adapts the myth of Drexciya from the recordings and artwork of Drexciya’s oeuvre and the song “The Deep” (Guardian, 2021). Yet, Solomon manages to bring a different perspective to this underwater city: a discourse that extends beyond what exists in historical discourse. Veronica Tello, in her *Counter-Memorial Aesthetics*, considers counter-memory not merely as reconstructing history or contestations of the past but as “a part in the construction of the future.” She adds, “The act of… counter- memorialization is not simply constitutive of ‘memory’, the fragile matter of recollection, it is the act of producing books, documentaries, films, literature and artworks that bring to the fore narratives that otherwise fall through the gaps of consciousness. This process shapes social memory, and is closely tied to history since it determines the ways in which communities and subjects imagine themselves vis-à- vis the past and, crucially, the future.” To Tello, how memories and historical records are thought about and presented will influence the formation of subjectivity and intersubjectivity (Tello, 2016, p. 34).

History is essential for understanding the human condition. Therefore, counter-memory and postmemory are essential to address the distortions and gaps in historical narratives, particularly those regarding the violence and trauma of marginalised communities. Solomon notes, “Without Yetu’s body, they could not transfer the History, and without the History, the wajinru would perish” (Solomon, 2019, p. 16–17). Solomon uses postmemories to create counter-memories, resisting the erasure of history and ensuring recovery of the lost black voices. This counter-perspective—tied to justice, recognition and healing - ensures that the voices of the historically oppressed or those affected by violence are heard and acknowledged. This process provides a more accurate understanding of history and facilitates catharsis for the victims and their descendants. Therefore, the paper’s contribution is to reassess historical narratives by including a polyphonic dimension of human experience.

## 2 Review of literature

The term postmemories was first used by Marianne Hirsch in *Family Pictures: Maus, Mourning, and Post-Memory* (1992–93). Later, Art historian Liss (1998) used the term to describe the effects of Holocaust photographs on post-Auschwitz generation. In 2012, Hirsch, in *The Generation of Postmemory: Writing and Visual Culture after the Holocaust*, refers to postmemories as the transfer of traumatic memories to the generation after without having to experience them directly. Hirsch also distinguishes “postmemory” from “rememory,” where the former refers to the relationship between descendants and the traumas of previous generations, and the latter is the traumatic repetition of individual repressed memories with a visible fixation on the past (Hirsch, 1993, p. 121). Postmemories finds its roots in memory studies and trauma theory and was initially understood in the context of the Holocaust. It has since then been studied and applied in a wider context (Creţan and Doiciar, 2022; Jeon, 2024), including virtual landscapes (Jones and Osborne, 2018). Postmemory has also been studied in the diasporic and migration fields (Baser and Toivanen, 2023; Runfors, 2023).

Counter-memory is associated with Michael Foucault’s essay *Nietzsche, Genealogy, History* (1971), and serves as a tool of resistance and the formation of an alternative historical perspective. Drawing from Foucault’s concept, Lipsitz (2001) applies Foucault’s counter-memory in a political and cultural context as a memory that challenges and resists the dominant historical narratives through the experiences of marginalized communities. Tachibana (1998), in *Narrative as Counter-Memory* (1998), discusses how minor narratives disrupt officially sanctioned hegemonic narratives and mythologies in Japan and Germany after 1945. Philosopher Medina (2011), studies Foucault’s counter-memory not simply as alternate memories that work as correctives, but rather as an energizing form of resistance. *Community Arts as Public Pedagogy: Disruptions into Public Memory through Aboriginal Counter-Storytelling* emphasizes the importance of centering and listening to Aboriginal voices. It also highlights the significant gaps in public memory and knowledge regarding the silences, omissions and misrepresentations that hinder the possibilities for fostering solidarities with Aboriginal people in addressing racialized exclusion (Quayle et al., 2016). Gusain and Smitha (2024) reassess the idea of a singular past and claim restoration and representation of marginalized memories into cultural memory.

The intersection of postmemories and counter-memories interrogates how this collective trauma is transmitted, processed, and transformed in the speculative, posthuman context. Despite the rich insights from existing research, a comprehensive study is needed on posthuman interferences, particularly in relation to narratives of postmemory and counter-memory in literature.

## 3 Materials and methods

This study employed a qualitative methodological framework, specifically narrative and discourse analysis, to examine Rivers Solomon’s novella *The Deep.* The aim is to gain insights into the lived experiences of individuals subjected to the traumas of forced migration during the transatlantic slave trade.

### 3.1 Theoretical framework

The paper closely analyses *The Deep*, questioning how the novella employs counter-memory to challenge the prevailing social memory. It also questions how counter-memory and postmemory, in the text, act as tools of resistance against trauma and hegemonic history. Finally, it questions how posthuman interventions challenge the boundaries between human and non-human agents in the construction of counter-memory and postmemory in the novella. By answering these questions, the paper employs postmemory as the theoretical framework to emphasize that the intergenerational trauma initiates a rebellion among the postgeneration and facilitates the creation of counter-memories through posthuman figurations within the narrative of the slave trade. Postmemory becomes the vehicle through which the wajinru community seek justice or emotional response to the injustices faced by their ancestors and the consequent emergence of counter-memories. To the wajinru community, history defines them as embodied in the title *The Deep* which symbolizes this new depth in the historical abyss (Solomon, 2019, p. 23).

## 4 Discussion

In the 1990s, Cathy Caruth’s trauma theory legitimized survivors’ testimonies, assessing them beyond the traditional metrics of reliability and accuracy; instead, they were conceived as an alternative system of knowledge about past historical events (Caruth, 1996). The concept of postmemory extends this authority to the subsequent generations of those affected, allowing them to articulate their experiences of inherited trauma. Such intergenerational testimony deepens the discourse surrounding trauma and memory, emphasizing the multifaceted nature of historical understanding. Therefore, understanding history in the light of memory reveals that history cannot be considered a static recollection of events but as one that is continuously reshaped across generations.

Stephen Frosh calls memory not a private thing but a socially mediated one: a cultural affair (2019, p. 162). He considers memories not simply as an individual experience but as influenced by a social context. Understanding Frosh’s in the light of Cathy Caruth’s trauma theory leads to Marianne Hirsch’s concept of Postmemory. In *The Generation of Postmemory*, she says, “Postmemory describes the relationship of the second generation to powerful often traumatic experiences that preceded their birth but nevertheless transmitted to them so deeply as to seem to constitute memories in their own right” (2008, 103). Though Hirsch talks of postmemory in the context of the Holocaust, she says that postmemory allows for an “evolving theoretical discussion about the workings of trauma, memory, and intergenerational acts of transfer… in numerous important contexts outside of Holocaust studies” (2008, p. 2). Postmemory is the cultural and collective trauma that the consecutive generation imbibe through stories, images and behaviors among which they grew. These memories are mediated not by recall but by imagination, creation and projection. Though these events are from the past, their effects continue to influence the present (2008, p. 107).

The trauma associated with postmemories becomes integral to the wajinru community, as this dimension enables the formation of counter-memories. *The Deep* reframes one of the most gruesome memories of the transatlantic slave trade, reimagining the murder of enslaved women as an escape from oppression and the formation of a utopian civilization (Solomon, 2019, p. 106). While colonial documentation attempted to erase the history and memory of enslaved and colonized people (Campa, 2017, p. 94), Solomon reimagines the experience of slavery, addressing the often superficial grasp of the historical context of the slave trade, particularly from the perspective of the descendants of enslaved populations. Within this perspective, she also incorporated mythical fragments alongside historical records to document her narratives. By subverting the mainstream narrative of the transatlantic slave trade and resisting the collective amnesia of the horrors of slavery, Solomon writes back to the center.

In the posthuman context, memories and postmemories are not restricted to humans (Knittel and Driscoll, 2017), and several posthuman interventions enable the creation of counter-memories. Solomon integrates posthuman interventions through Yetu’s character, portraying her as a vessel who carries the memories of her people. The wajinru, as hybrid beings embodying both human and fish forms, emerge from the lineage of enslaved Africans and have evolved into something beyond the conventional human understanding. The themes of postmemories are deeply felt in Yetu’s internal conflict, which transcends a singular, unilateral human-centric perspective of trauma. This represents a posthuman existence where memory and identity are both individual and collective experiences. This allows the posthuman beings whose human bodies and minds are embedded with memories of slavery and trauma to undergo catharsis in their new posthuman existence.

### 4.1 Inherited trauma and postmemories in *The Deep*

In *The Deep*, the concept of postmemory plays a pivotal role in shaping the characters’ experiences and identities. In the underwater civilization, Yetu, the historian, is described as “a vessel for the ancestor’s memories” (Solomon, 2019, p. 7) and is tasked with the monumental responsibility of safeguarding the social memory of the community. These memories are meticulously transmitted across generations of historians and are shared with the community during the annual day of Remembrance, an event dedicated to reconnect the wajinru with their historical roots. However, these memories fade within a few weeks due to wajinru’s biological predisposition to forgetfulness. Nevertheless, it is the historian’s duty to remember and carry forward the memories, presenting them again the following year, thereby ensuring their survival. Therefore, the role of Yetu in the novella is indispensable for showing “the immediate and visceral pain inherent in passing down past trauma” (Solomon, 2019, p. 107).

The practice of memory transfer, as portrayed in the novella, dates back to previous historians who “had spent their days roaming the ocean to collect the memories of the living wajinru before they were forgotten. Such a task ensured that the historian understood who was best suited to take on the role after their own death” (Solomon, 2019, p. 8). Yetu’s memories contain the trauma from the transatlantic slave trade, particularly the memory of the “two-legged bodies”- their human ancestors - who had been discarded into the sea many years ago and these drowned first mothers who gave birth to the early wajinru. However, postmemories were transmitted differently in the novella, where “they did not remember in pictures, nor did they recall exact events, but they knew things in their bodies, bits of the past absorbed into them and transformed into instincts” (Solomon, 2019, p. 11). That is why, though these wajinru have never been to America, never seen the plantation sites, nor been slaves, wajinru instinctively avoid shallow waters where they might encounter two-legged (white) surface dwellers.

In the context of postmemory, the memory is not characterized by direct recollection of the event but emerges as an evocative sensation or feeling: the intangible “something” that is communicated through the parent’s retellings and is subject to distortions and vagaries due to lack of direct experience of the trauma. This inhabited memory, which comes from someone or somewhere creates a lurking sense of threat, injury and loss that shapes the individual’s way of existence in the world (Frosh, 2019, p. 160). Yetu, as a historian, held on to the weight of 600 years of pain. This meant “all the memories of those who have come before, they lived inside me. Real as flesh. I remembered them like they were my own. I walked inside them” (Solomon, 2019, p. 64). The “traumatic transfer” (Hirsch, 2008, p. 108) exacerbates the ancestors’ suffering and its impact on the living. Although not all memories were suffering, there were times of joy, happiness, and decades of bounty, but the sad moments were totalizing (Solomon, 2019, p. 26). Despite her attempts to swim away from the pains of the past, she recognizes that no matter how far she swims, the past always haunts her (Solomon, 2019, p. 46). Yetu’s role as a historian is pivotal in shaping her posthuman identity and as Misztal (2003, p. 33) notes, identity and memory are interdependent, as identity is rooted in memory, and what is remembered is shaped by the assumed identity. Most of the time, the postmemories are enmeshed with her own memories, and it strikes Yetu with a veil of moral ambiguity as a historian where her collective and individual destinies have always been entangled.

The intergenerational and transgenerational postmemories enable individuals and collective groups who have never experienced them to still feel a past event or place as meaningful and emotionally significant (Hirsch, 2012). Hoffman states, “the paradoxes of indirect knowledge haunt many of us who came after. The formative events of the twentieth century have crucially informed our biographies, threatening sometimes to overshadow and overwhelm our own lives. But we did not see them, suffer through them, experience their impact directly. Our relationship to them has been defined by our very “post-ness” and by the powerful but mediated forms of knowledge that have followed from it” (Hoffman, 2004, p. 25). The trauma contained in the memories of the wajinru community are those of the ancestors but is passed on from one historian to another and now to Yetu. She grapples with the traumas in the postmemory, which often drive her toward self-destruction and suicidal impulses. She feels “adrift in a memory that wasn’t hers” and considers death to relieve the burden for the next historian so that they need not “harvest the ancestor’s rememberings from Yetu’s mind” (Solomon, 2019, p. 6–7). The novella goes on to say, “the rememberings were always drawing her backward into the ancestors’ memories—that was what they were supposed to do—but not at the expense of her life” (Solomon, 2019, p. 6).

The selection of historians by the predecessors is based on the mind’s capacity to be electro-sensitive large enough to host and transmit the rememberings in the future (Solomon, 2019, p. 8). Although there is no clear account of the former historian’s coping mechanism for the trauma associated with these memories, Yetu’s psychological state visibly deteriorated over time. Her initial years as a historian were extremely difficult, and she struggled to do basic things like holding her bowels for more than a few minutes (Solomon, 2019, p. 21). And with each passing year, her condition worsened, impairing her ability to distinguish the past rememberings from her current lived experiences (Solomon, 2019, p. 6). Solomon recounts, “Sometimes, the rememberings took precedent over everything else, even over the survival of the present” (Solomon, 2019, p. 28). The annual ceremony of Remembrance only worsened her plight, compelling her to relive wajinru’s entire history during the transfer process. To Solomon, “Yetu was becoming an ancestor herself. Like them, she was dead, or very near it” (Solomon, 2019, p. 9).

During the ceremony of the Rememberance, when all the wajinru experience their history, it culminates in an epiphany regarding their connection to the two-legged ancestors who suffered under the oppressive hand of white slave traders. As “the wajinru understood how related they were to the two legs, the remembering changed… They were all now one of the floating dead bodies. The wajinru felt the deadness like it was their own” (Solomon, 2019, p. 28). They realized that the two-legged were their ancestors who suffered in the hands of the white slave traders. But “the wajinru experienced the rememberings like they were living out their own memories” (Solomon, 2019, p. 26). This realization, brought forth through the rememberings, deepens their emotional response, fueling a sense of rage and sorrow that culminates in a storm in the sea and affects the surface dwellers at the novel’s climax.

In the novella, Yetu’s momentary relief from her people’s history after its transfer to the wajinru, fosters a quest for permanent freedom and prompts her to leave the wajinru and their history without transferring it back to herself. While the other wajinru are stuck in their history, Yetu’s attempt to escape from her overwhelming pain and trauma by retreating to the surface reveals a futility in her endeavor; she discovers that there is nothing she can do to take the pain away from her. She finds herself trapped in a relentless cycle of anguish, as indicated by the narrative: “the place she’d gone from was a world of pain, and there was no distance she could swim where that past would not haunt her” (Solomon, 2019, p. 46). Ultimately, despite Yetu’s struggle to live with the pain, she could not escape her history and must endure it for the sake of her people because without their history, they would be empty (Solomon, 2019, p. 25). This tension underscores the pervasive impact of historical trauma on both individual and collective identity through postmemories.

While Yetu’s postmemories consume a significant portion of the book, Oori’s memories are also crucial in the novella. Oori’s historical dislocation and loneliness due to these historical gaps, make her incapable of thinking beyond “the screams of the last remaining specimens of her people” (Solomon, 2019, p. 81). Unlike Yetu, who tries to escape from her history, Oori actively seeks the memories that were not passed down to her. To her, knowledge of her history is a key to understanding herself. She desperately attempts to fill her historical void, which signifies her struggle for identity, leaving her lost in the world due to the lack of postmemory continuity. She expresses her urgency, saying, “I need to take a pilgrimage back to my homeland before it gets worse. I need to protect some of the fixtures, tend to the grave sites, lest they all vanish and the place I’m from become truly dead” (Solomon, 2019, p. 82). She also emphasizes the necessity of the homeland, asserting it as a place “that means something because of its history.” She adds, “I know you have a complicated relationship with the past. I do too. But if I do not protect what is left …, I will have no homeland. It will just be another place” (Solomon, 2019, p. 83).

Frosh describes postmemory as an imaginative process that diverges from conventional memory, as it is not formed through direct recollection of lived experiences, but rather through a meticulous process of construction or formation (2019, p. 162). In this context, *The Deep* illustrates how postmemory serves as a powerful tool for reconstructing or reimagining the past. This process not only responds to trauma inflicted on the characters but also navigates and reinterprets their collective and individual experiences in a way that shapes their identities. Therefore, a vigorous resistance against oppression arises in the wake of shared trauma as the characters draw on their postmemories to challenge and redefine their realities.

### 4.2 Counter-memory as a space for resistance in *The Deep*

Michel Foucault originally coined the term “Counter-Memory,” or contre-mémoire, in his essay *Nietzsche, Genealogy, History* (1971), which was published in English in *Language, Counter-Memory, Practice: Selected Essays and Interviews by Michel Foucault*, edited by Donald F. Bouchard. Foucauldian interpretation of counter-memory is the forging of a “totally different form of time” (1971, p. 385) and registers a forgotten record of oppression and resistance. It opposes the idea of history as “reminiscence or recognition” or “continuity or representative of a tradition” or “true knowledge” (Foucault, 1971, p. 160). Foucault asserts that when institutions of power systematically censor the expressions and ideas of other groups, these ideas become subjugated knowledge, contrary to the projected knowledge. Historians align themselves with this dominant knowledge and fail to see particular blocks in historical narratives. Therefore, by critiquing this projected knowledge, we unearth and rediscover the subjugated knowledge. When this rediscovered knowledge is used to modify our interpretation of history, it constructs a counter-memory from the known projected knowledge. Through this, indigenous and marginalized cultures reclaim their cultural knowledge and rewrite history from their vantage points. Veronica Tello notes that counter-memory prioritizes the hoarding of histories, archives, and experiences (2022, 399); however, it is not a blind rejection of history but rather its reconstitution.

Although Michel Foucault is most often credited with the term counter-memory, many other thinkers have also explored this concept across diverse fields. Toni Morrison (2004, 2007) works, particularly *Beloved* and *Playing in the Dark,* recounts stories of black slaves distorted by dominant white narratives. Other notable works like Benjamin (1990) *Theses on the Philosophy of History*, Agamben (1999) *Remnants of Auschwitz: The Witness and the Archive*, Trouillot (2015) *Silencing the Past: Power and the Production of History* and Ricœur (2010) *Memory, History, Forgetting* also engage with the complexities of recovering and reclaiming suppressed histories. They infer counter-memory as a mode of resistance against historical amnesia and the intentional blurring or distortion of historical accounts. It does not only deal with lost histories but also engages with the power dynamics inherent in constructing history and forming collective memory by questioning what the nation is allowed to remember.

Counter-memory refers to the “practice of memory formation that is social and political, one that runs counter to the official histories of governments, mainstream mass media, and the society of the spectacle” (Animate Projects, 2012). Demos in *Animate Projects*, describes counter-memory as the collective practice of relearning the forgotten, suppressed, and excluded histories, which in turn function as an act of political subjectification. It can be understood as a conceptual framework and a methodological approach aimed at mobilizing memories and struggles of the marginalized in response to violence and systemic dispossession. Thus, counter-memories are depicted as “grappling with two temporalities: that which has been diminished or erased and that which is monumental. By mediating the tensions of these temporalities, it is seen to energize social movements—counter-memory is cast as a critical catalyst of social justice” (Tello, 2022, p. 391).

Through Yetu’s memories, we learn the grim historical reality of African slaves during the transatlantic slave trade, specifically the practice of discarding them overboard. The older wajinru find new ones emerging frequently over the years - a practice followed over an extended period. One particular story centers on a young wajinru who, when found, was blind, deaf and bore multiple scars on her body, symbolizing the brutal conditions faced by the slave workers on the ship. This young wajinru hears the cries of the malnourished, suffering captives on the lower decks of the ships, who have been robbed of their homes and stolen from their families. Their lives are no longer their own and belong to their masters on the ship (Solomon, 2019, p. 41). The wajinru represents the descendants of the two-legged confined in the lower decks rather than those on the above deck. This lower deck holds the slaves who are deemed disposable and as a drain on resources during the journey.

To Solomon, “History was not an imagining, not just stored electrical pulses. They were people who’d lived. Who’d breathed and wept and loved and lost” (Solomon, 2019, p. 18). She retells a fictional but consequential story of “white supremacist violence” (Solomon, 2019, p. 108) - the enslavement, torture and death of the African slaves during the slave trade. During the ceremony of the Remembrance, Yetu is in overwhelming pain as she feels every wajinru who lived before her possess her in that moment: “they gnashed, they clawed, desperate to speak,” (Solomon, 2019, p. 22) and tell their story of pain and violence. This desperation, silence and anger catalyze a counter-memory that Solomon meticulously articulates throughout the novella, reflecting the deliberate attempt to address the erasure of African American histories. Thus, the act of remembering and reclaiming, through the experiences of the wajinru, is crucial in creating and recollecting the histories of African slaves.

During the annual ceremony of Remembering, Yetu “wanted the people to remember how she remembered. With screams. She had no wish to transform trauma to performance” (Solomon, 2019, p. 11). Their history, due to its brutality, seemed hard for the wajinru to believe at first. They wondered if the first wajinru really saw bodies being thrown overboard into the sea and left to drown (Solomon, 2019, p. 86). Yetu, as a historian, had “dealt with death every day during her rememberings” (Solomon, 2019, p. 14), and the trauma was not new to her. But the wajinru, brimming with the history of their past and unable to hold their anger, say, “In the old days, when we discovered a ship that threw our ancestors into the sea like refuse, we sunk it. Now we will sink the world” (Solomon, 2019, p. 86). As more and more memories are being transferred to them, their rage grows. Consequently, they begin a war with the two-legged creatures (Solomon, 2019, p. 93).

When the wajinru were brimming with their fill of history, they felt “restless energy build up in us, wanting to explode. Our amaba used to call this spoiling for a fight. And it’s true, we always were, always still are. We do not know what to do with quietness, with peace. Life in the deep has never suited us” (Solomon, 2019, p. 87). When historical narratives saddened other communities, the wajinru community felt only a burning anger. Therefore, anger emerges as a predominant emotion, providing a sense of purpose and belonging to them (Solomon, 2019, p. 88). This proactive stance presents an opportunity to fight back, a form of resistance: an emotional response to the long-standing accumulation of anger and pain from the injustices inflicted upon their ancestors. The wajinru’s anger simmers beneath the water, building like a storm set to destroy those on the surface. The two-legged were not new to storms, but this storm was one like never before. It was a “war between the wajinru and the surface dwellers,” and “they were so lost in it, they were taking their grief out on the whole world” (Solomon, 2019, p. 84–85). The wajinru’s shared fury strengthened them, and they continued to create a tidal wave that lifted them high above the land. They say, “We send endless waves of salt water onto the land, flooding the whole earth. This is only our first assault” (Solomon, 2019, p. 94). This militant uprising by the wajinru is a form of self-sought justice, a catharsis. Yetu recounts a particular memory when one wajinru heads upward toward the surface to verify if their history was true and if the two-legged beings existed, but when the wajinru could not heave in oxygen on the surface, she grew unconscious. When the two-legged species came to grab the wajinru, she retorted and bit their throats until they died. This is a consequential response to the rage and anger of the rememberings. To them, “This truth, that two-legs were cruel and unusual, was the most important lesson of the History” (Solomon, 2019, p. 87).

To Yetu’s amba, the essence of survival serves as a more significant tribute to their ancestors than mere adherence to tradition (Solomon, 2019, p. 99). The Western dichotomy between life and death is undone with a life-death continuum, reflecting the resistance and resilience in the new utopian civilization. When Yetu finally returns to the other wajinru, to prevent them from terminally destroying the whole earth, she says, “despite the wajinru being cradled in the ocean’s depths, their turmoil had affected the whole sea, extending up to the surface where the storm raged” (Solomon, 2019, p. 97). This highlights how the collective trauma inherited through postmemories spurs the wajinru to engage in this battle (Solomon, 2019, p. 92). The history of wajinru’s survival and their fight through the raging storm serves as a counter-memory to the history of the enslaved Africans. Through this portrayal, Solomon deconstructs the history of colonization and rewrites alternative history from a new vantage point, showing how complicated and fragmented the slave past truly was. Solomon weaves a counter-memory of survival and strength in the face of historical oppression.

The pain associated with postmemories is integral to the wajinru community, as this dimension of pain provides both a purpose to avenge and a sense of identity. To Solomon, regardless of the magnitude of the trauma embedded in history, individuals should not willingly cut themselves out of it. It is seeking and embracing this weight of the past that makes a wajinru truly a wajinru. The discourse of life after death, their survival and resistance serve as the counter-memory to the deaths of slaves during the transatlantic slave trade. And it is the children born from the deceased slaves who give voice to the slave community and emerge as narrators of the tale of their ancestors. The storm at the end of the novella manifests the unrest felt by the wajinru, who embody the collective rage over the historical atrocities inflicted upon their ancestors, an interpretation of a form of resistance against the hegemonic power structures.

Solomon articulates a counter-memory narrative that recontextualizes the experience of the slave descendants, challenging the hegemonic discourse that the blow of colonization is deemed to keep them forever subjugated. The narrative of subjugation and relentless endurance is replaced with a new narrative that portrays communal existence, resilience, and healing. All these are portrayed to be just as vital as survival. Solomon says, “they live lives of togetherness, in the present. That the many of them who started out their lives in loneliness and solitude, they must put it away, and remember where we are now. Together. Safe” (Solomon, 2019, p. 44–45). This recontextualization underscores not only the enduring impact of past abuses and trauma but also the transformative potential of community and shared experience in fostering a sense of safety and belonging: a journey toward catharsis.

Solomon believes “forgetting was not the same as healing” (Solomon, 2019, p. 22). Therefore, Solomon notes the need for counter-memories by describing the need for “varied interpretations of History” as “what had always seemed certain to Yetu wasn’t so immutable. The living put their own mark on the dead” (Solomon, 2019, p. 101). Such recovery of the past and its recontextualization create alternate histories essential for constructing individual and collective identities (Abdulaal, 2016, p. 1397). This makes the subjugated community resist the omissions and distortions of official histories by bringing back lost voices and forgotten experiences. Hartman (2008, p. 11) calls this technique as “critical fabulation” which, “is not to give voice to the slave, but rather to imagine what cannot be verified, a realm of experience which is situated between two zones of death—social and corporeal death—and to reckon with the precarious lives which are visible only in the moment of their disappearance” (2008, p. 12). Solomon, through the narrative of Yetu and the other wajinru, effectively weaves these historical and emotional experiences into the fabric of the story to foreground the anguish of the characters.

As Lipsitz says, the concept of “counter-memory” could also be the practice of deliberately forging revisions in historical narratives, particularly through the perspectives and mythic imaginations of communities that experience ongoing oppression. With the incorporation of creatures like wajinru and the myth of Drexciya, Solomon has moved to a new dimension in the revival of ancestral history by infusing mythical imagination into her historical narrative. Counter-memories to Davis (2000, p. 149) is a creative re-thinking of the forgotten or excluded ideas, ultimately facilitating the emergence of new potential within the present existence. This ability to put fragments together and reclaim history, flesh and memory, is essential for survival (Goellnicht, 1997, p. 352). Recovering a memory from a fictional standpoint offers a new perspective to explore a particular memory. To Lipstiz, “Story-telling that leaves history to the oppressor, that imagines a world of desire detached from the world of necessity, cannot challenge the hegemony of dominant discourse. But story-telling that combines subjectivity and objectivity, that employs the insights and passions of myth and folklore in the service of revising history, can be a powerful tool of contestation” (Lipsitz, 2001, p. 212–213). Therefore, counter-memory integrates aspects of myth and history while retaining an enduring suspicion toward both categories (Lipsitz, 2001, p. 213).

### 4.3 Posthuman interventions for rewriting history

The novel reimagines historical archiving through the lens of the nonhuman. This posthuman reconfiguration of memory emphasizes alternative ways of knowing—communal, embodied, and affective—over traditional, hierarchical, and exclusionary frameworks. Conventional historical archives are predominantly anthropocentric, privileging written documentation over oral narratives, bodily experiences, and nonhuman forms of memory. In contrast, *The Deep* reimagines the notion of the archive by centering wajinru’s bodily and sensory interaction with history. Their memories do not reside in textual form; instead, they are embedded within living entities and transmitted through deep feeling, highlighting a fluid and dynamic approach to memory rather than rigid, static documentation.

In a humanist landscape where memory has predominantly been a human attribute, the novella expands memory (along with postmemories) into new spheres by including nonhuman wajinru. The novella decenters human subjectivity and forms a human-nonhuman assemblage, highlighting the interconnection of the human, nonhuman, and the environment. The nonhumans are not merely passive carriers of historical memory; they actively participate in forming counter-memories that reshape collective understanding. This exploration of memory integrates the experiences of both humans and nonhumans, enabling catharsis within their posthuman existence. In this context, the ocean serves as a posthuman memory-scape, characterized by its fluidity and collectivity, effectively subverting linear conceptions of time. The novella thus serves to reconnect humans with different bodies, histories, and ecologies that humanism has violently severed.

By positioning Yetu as a narrative vehicle, the text critiques hegemonic historiographies and underscores the multiplicity of voices in recounting historical experiences. The incorporation of characters like Yetu - a marginalized female, homosexual, hermaphrodite, and nonhuman descendant of a Black slave- challenges the archetypal white, male, historian figure to convey historical insights highlighting the intersectionality of history and identity. These multifaceted characters not only propel the plot but also invite readers to reconsider the power locations of subject-position—whose stories are told and who gets to tell them. This cartographic account through wajinru figurations is an active pursuit of alternative visions of the subject (Braidotti, 2013, p. 164). The novella also undoes the Western dichotomy between life and death by incorporating wajinrus who exist in a life-death continuum. To Çimen (2024), “decay and regeneration are entangled processes which… testifies to a posthumanist rethinking of life in general, to the monistic perspective that what is past and decayed is… in connection with the present and the future, and that life does not rest on the idea of purity or segregation but instead flows through relational becomings of all forms of earthly agencies” (2024, p. 376).

## 5 Conclusion

Posthumanism embarks on a new agenda that departs from Eurocentric universalism to a post-nationalist perspective, signaling the decline of Eurocentrism (Braidotti, 2006, p. 52–53). Benhabib (2008) in *Another Cosmopolitanism* questions Europe’s role as the epicenter of transformation; therefore, a posthuman framework diverts from this Eurocentrism and invites new ways to think about the interplay between humans, non-humans, and the material world. Solomon’s approach offers a fluid, fragmented, and non-human perspective on past injustices and their associated trauma. “They were all now one of the floating dead bodies. The wajinru felt the deadness like it was their own” (Solomon, 2019, p. 28), indicating the posthuman stance of the nonhuman and human becoming one. The wajinru refuse the politics of their historical negation, articulating a subjectivity that is in part with multiple beings. This pluralistic and relationalist perspective, informed by post and counter-memories, reconceptualizes life within a posthumanist framework. It also highlights how memory and resistance transcend human experience by integrating non-human actors, technologies, or even ecological systems. Such an expansion enriches our understanding of healing and resistance, broadening the parameters of what can be included in these dynamics.

Yosef Hayim Yerushalmi, in *Jewish History and Jewish Memory*, argues that although the danger of remembering too much could be a refusal to move on from things, he regards forgetting as an even greater terror than the terror of remembering. He says, “Is it possible that the antonym of ‘forgetting’ is not ‘remembering,’ but justice?” (Yerushalmi, 1989, p. 117). This notion emerges in the narrative of Yetu, where a persistent desire to forget the horrors of the past clashes with the necessity to remember in order to heal and reclaim one’s identity both as an individual and as a collective. Therefore, this duality is crucial to achieve justice. Certain memories are passed down from one generation to the next in an attempt to prevent their erasure and ensure they are not forgotten forever. Solomon, in the novella, gives voice to these historically marginalized communities and effectively brings these crucial memories to the fore.

Solomon’s creation of Yetu is a medium of postmemory who act as storage units for cultural artefacts. Postmemories are crucial for understanding Yetu’s history, and she carries the ancestors’ memories without necessarily experiencing the original event herself. Lipsitz contends this knowledge of history as essential, noting, “we may never succeed in finding out all that has happened in history, but events matter and describing them as accurately as possible can, at the very least, show us whose foot has been on whose neck” (1990, p. 212). Yetu lives with the echoes of her ancestors’ violence and suffering, which haunt her, and her initial response is to flee from them in pursuit of freedom. Ultimately, she realizes, “If freedom brought loneliness, emptiness, what was the point?” She realized that “Nothingness was a fate worse than pain” (Solomon, 2019, p. 85), and despite the pain and trauma from the postmemories, it shapes her identity: “Pain filled her, but so did knowledge, beauty… All of these things had made Yetu.” The postmemories, albeit painful and ugly, were hers. Without it, she was empty (Solomon, 2019, p. 98). Yetu’s history consumed her in devastating ways, but freedom from it left her empty and at a loss of identity.

Though Solomon uses actual historical events and the injustices inflicted on the enslaved, she transcends the purely historical narrative by incorporating mythical elements. This alternative approach to archiving historical memory through the integration of fictional narratives reimagines the lived experiences of the enslaved beyond the confines of conventional archival documentation. The counter-memories created honors the legacy of resistance against their historical subjugation, serving as a vital mechanism for articulating repressed emotions and fostering collective catharsis within the community. In the context of Afrofuturism, these counter-memories also catalyze the formation of counter-futures, which are not only a reclamation of their past but also a venture into new reimagined futures, different from the ones the colonizers intended for them to have. Therefore, “to establish the historical character of black culture, to bring Africa and its subjects into history denied by Hegel et al., it has been necessary to assemble countermemories that contest the colonial archive…” (Eshun, 2003, p. 288).

Solomon draws a dualistic image through Yetu and Oori, to emphasize the significance of identity and historical consciousness. Solomon says, “History was everything. Yetu knew that. But it wasn’t kind” (2019, p. 66) and without the knowledge of their history, they felt out of place and out of time (2019, p. 57). In contrast, Oori feels so lost being the last representative of the Oshuben tribe and laments, “I would take any amount of pain in the world if it meant I could know all the memories of the Oshuben. I barely know any stories from my parents’ generation. I cannot remember our language. How could you leave behind something like that? Does not it hurt not to know who you are?” (Solomon, 2019, p. 64), signifying a profound yearning for ancestral continuity and historical roots. By the novella’s end, it is evident that Oori’s lineage is linked to the enslaved individuals trafficked during the transatlantic slave trade: individuals with no cultural memory or ancestral ties in the new place-less and people-less context. Though at first, it may seem that only the lives of those thrown overboard seem lost, even those who survived and lived had no history of their own and were lost with no sense of belonging. Both Yetu and Oori grapple with history, and its knowledge was inevitable for both.

In *The Deep,* the narrative weaves characters’ memories within a broader collective memory, influencing their sense of identity and selfhood. The characters constantly grapple with their forgotten histories and strive to reclaim their voices to tell the stories of their ancestors. Solomon explores the intersection of postmemory and counter-memory by emphasizing that trauma is not just a personal experience but also a collective one that unites communities across generations and species in ways that transcend individual lives. Through this interplay, Solomon gives voice to the marginalized and forgotten community forming counter-memories to hegemonic discourses. This dynamic emphasizes the importance of remembering, reclaiming, and reimagining history as essential for the catharsis of both individuals and communities.

## Data Availability

The original contributions presented in the study are included in the article/supplementary material, further inquiries can be directed to the corresponding author.
